# The Tapeworm

**Published:** 1879-05

**Authors:** 


THE TAPEWORM.—In a recent German publication we are told that black oxide of copper is the surest and best cure for tapeworms. It is given in pills made according to the following formula:

| | Grammes |
|---|---|
| Cupri Oxydati nigri . . . | 6 |
| Calcariæ carbonicæ . . . | 2 |
| Boli albi lævigatæ . . . | 12 |
| Glycerin . . . . . | 10 |

Make 120 pills. Take 2 four times daily.

It is said to have this disadvantage, that the patient is denied the pleasure of exhibiting his tormentor.—*Chemist and Druggist.*

---

In a sketch of the life of the late Dr. George B. Wood it is stated by his nephew, Dr. H. C. Wood, that he never, even during his student life, witnessed the complete process of parturition. According to his own statement, the nearest he ever came to seeing a child born was when he was hastily summoned, in the night, to a fashionable boarding-school, to see one of the pupils who was said to be suffering from a colic. Placing his hand upon her abdomen, during a paroxysm, the true condition of affairs flashed upon him, when, saying to the anxious principal, "Send for Dr. Hodge," he turned upon his heel and fled.—*Cin. Lancet and Clinic.*

